# FTIR Microspectroscopy Coupled with Two-Class Discrimination Segregates Markers Responsible for Inter- and Intra-Category Variance in Exfoliative Cervical Cytology

**DOI:** 10.4137/bmi.s592

**Published:** 2008-03-25

**Authors:** Michael J. Walsh, Maneesh N. Singh, Helen F. Stringfellow, Hubert M. Pollock, Azzedine Hammiche, Olaug Grude, Nigel J. Fullwood, Mark A. Pitt, Pierre L. Martin-Hirsch, Francis L. Martin

**Affiliations:** 1 Biomedical Sciences Unit, Department of Biological Sciences, Lancaster University, Lancaster, U.K; 2 Lancashire Teaching Hospitals NHS Trust, Preston, U.K. and; 3 Department of Physics, Lancaster University, Lancaster, U.K

**Keywords:** biomarker, cervical cytology, Fourier-transform infrared microspectroscopy, high-grade, low-grade, principal component analysis

## Abstract

Infrared (IR) absorbance of cellular biomolecules generates a vibrational spectrum, which can be exploited as a “biochemical fingerprint” of a particular cell type. Biomolecules absorb in the mid-IR (2–20 μm) and Fourier-transform infrared (FTIR) microspectroscopy applied to discriminate different cell types (exfoliative cervical cytology collected into buffered fixative solution) was evaluated. This consisted of cervical cytology free of atypia (i.e. normal; n = 60), specimens categorised as containing low-grade changes (i.e. CIN1 or LSIL; n = 60) and a further cohort designated as high-grade (CIN2/3 or HSIL; n = 60). IR spectral analysis was coupled with principal component analysis (PCA), with or without subsequent linear discriminant analysis (LDA), to determine if normal *versus* low-grade *versus* high-grade exfoliative cytology could be segregated. With increasing severity of atypia, decreases in absorbance intensity were observable throughout the 1,500 cm^−1^ to 1,100 cm^−1^ spectral region; this included proteins (1,460 cm^−1^), glycoproteins (1,380 cm^−1^), amide III (1,260 cm^−1^), asymmetric (ν_as_) PO_2_^−^ (1,225 cm^−1^) and carbohydrates (1,155 cm^−1^). In contrast, symmetric (ν_s_) PO_2_^−^ (1,080 cm^−1^) appeared to have an elevated intensity in high-grade cytology. Inter-category variance was associated with protein and DNA conformational changes whereas glycogen status strongly influenced intra-category. Multivariate data reduction of IR spectra using PCA with LDA maximises inter-category variance whilst reducing the influence of intra-class variation towards an objective approach to class cervical cytology based on a biochemical profile.

## Introduction

There remains a need for objective approaches to identify cellular changes that may be applied for disease diagnosis (Walsh et al. 2007a). Fourier-transform infrared (FTIR) microspectroscopy may generate an IR “biochemical signature” unique to cell types. This has been applied to a range of tissues including the cervix (Cohenford and Rigas, 1998; Hammiche et al. 2007; Tobin et al. 2004; Walsh et al. 2007a; 2007b), colon (Argov et al. 2002; 2004), prostate (Gazi et al. 2003; 2004; Malins et al. 2003), skin (Tfayli et al. 2005), oral mucosa (Tobin et al. 2004), stomach (Fujioka et al. 2004), aorta (Bonnier et al. 2006) and lymphocytes (Andrus and Strickland, 1998). With IR analysis, a “susceptibility-to-adenocarcinoma signature” according to zonal location in the prostate was proposed (German et al. 2006a). Cells located in the stem cell niche of the cornea appear to possess identifying IR spectral characteristics (Bentley et al. 2007; German et al. 2006b). IR microspectroscopy may be applied to monitor cell cycle (Hammiche et al. 2005) or response to toxic insult (Barber et al. 2006).

Exfoliative cytology is used for screening of precancerous cervical lesions and this involves isolating cells that reside in the transformation zone of the cervix. Bordered by the ectocervical zone and endocervical canal, exfoliative sampling of cytology from this region generates a specimen containing two different cell types. This is because the endocervical canal is lined by tall columnar mucous-secreting epithelial cells whereas those derived from the ectocervix are thick stratified squamous epithelial cells (Chiriboga et al. 1998; Young and Heath, 2001).

Identification of pre-malignant stages of cervical cancer from routine screening has led to dramatic reductions in the incidence of this disease (Quinn et al. 1999). Current cytology consists of subjectively assigning categories to smears based on visual identification of the level of cellular dyskaryosis. Categories of pre-malignancy are assigned as cervical intra-epithelial neoplasia (CIN). These are sub-typed as CIN1, CIN2 or CIN3 dependent on the severity of cellular atypia. The Bethesda classification is also used and employing this method, severity of dyskaryosis is classed as either low-grade squamous intraepithelial (LSIL) or as high-grade squamous intraepithelial (HSIL). CIN1 describes a low-grade lesion with CIN2/3 indicating the presence of a high-grade lesion. Upon detection, individuals with CIN1 cytology are closely monitored to detect progression to CIN2/3 or regression to normal cytology (Ostor, 1993).

Exfoliated cervical cells are now most commonly transferred into a liquid fixative for subsequent preparation of a Pap smear rather than smeared onto a slide at the point of collection. This type of cytology, referred to as liquid based cytology (LBC), has the added advantage of improved sensitivity and a reduction in the occurrence of inadequate smears (Karnon et al. 2004). However, any improvement using LBC over the conventional smear is still being validated (Davey et al. 2006).

Biomolecules absorb in the mid-IR (2–20 μm) giving rise to vibrational spectra. This allows the measurement of a range of cellular components including protein conformation, DNA, RNA, lipids and glycogen content; these are often characteristic of cell type and/or pathology (Walsh et al. 2007a). The main IR absorbance bands are amide I (1,650 cm^−1^), amide II (1,550 cm^−1^), amide III (1,260 cm^−1^), asymmetric phosphate (ν_as_PO_2_^−^; 1,225 cm^−1^), symmetric phosphate (ν_s_PO_2_^−^; 1,080 cm^−1^) and glycogen (1,030 cm^−1^) (Hammiche et al. 2005; Walsh et al. 2007b; Walsh et al. 2008). Alterations in absorbance bands (i.e. shape, shifts and/or intensity changes) are indicative of intracellular alterations. The amide I band is mainly due to the _ν_(C = O) bond whereas the amide II peak associated with _δ_(N-H) and _ν_(C-N) protein bonds (Goormaghtigh et al. 2006; Mantsch and Chapman, 1996).

Attenuated total reflection-FTIR (ATR) microspectroscopy facilitates the interrogation of specimens with an aperture of ≈250 μm × 250 μm. This gives a high signal-to-noise ratio (SNR), with fast acquisition of IR spectra (Walsh et al. 2007a; 2007b). Spectroscopic studies generate large data sets that subsequently require computational data reduction approaches in order to identify variance e.g. principal component analysis (PCA) (Walsh et al. 2007a). With PCA, each spectrum is converted into a single point (or score) in *n*-hyperspace and groups of scores may be viewed in 3-dimensions rotated on principal components (PCs). Scores are spatially separated proportional to spectral similarity (Walsh et al. 2007a; 2007b). We investigated whether exfoliated cytology specimens in LBC fixative, which had been conventionally categorised as normal (n = 60), low-grade (n = 60) or high-grade (n = 60) could be segregated following ATR microspectroscopy and multivariate analysis.

## Materials and Methods

### Study populations

Specimens of exfoliative cytology (n = 180) were collected into SurePath fixative (TriPath Care Technologies, Burlington, NC, U.S.A.). Informed consent to obtain specimens for research was obtained (LREC No. 05/Q1308/2; Preston, Chorley and South Ribble Ethical Committee). A representative sample of all exfoliative cytology specimens was categorised by an expert pathologist employing conventional visual methods. Specimens of exfoliative cytology which had been conventionally categorised as normal (n = 60), low-grade (n = 60) or high-grade (n = 60) were obtained for the purposes of this study.

### LBC preparation for ATR microspectroscopy

From an original total volume of approximately 5 ml LBC specimen containing exfoliative cervical cytology, 1 ml of suspension was removed. Previous experiments demonstrated that for all specimens this generated a sufficient concentration of cellular material for the purposes of the assay. Exfoliated cytology specimens were centrifuged at 1500 rcf for 5 min. Supernatant was aspirated carefully leaving behind the residual cell pellet in ≈200 μl of fixative. The pellet was re-suspended in the remaining fixative and the cellular suspension was applied to 1 cm × 1 cm Low-E glass microscope slides (Kevley Technologies, Chesterland, OH, U.S.A.); these were allowed to dry in a sterile environment overnight. The microscope slides with adhered cellular material were then transferred to a dessicator until analysis with ATR microspectroscopy.

### ATR microspectroscopy

IR spectra were acquired using the Bruker Vector 22 FTIR spectrometer with Helios ATR attachment containing a diamond crystal (Bruker Optics Ltd, Coventry, U.K.). Using a CCTV camera attached to the ATR crystal, 10 random points were interrogated. Data were collected in ATR mode and spectra (8 cm^−1^ spectral resolution, co-added for 32 scans) were converted into absorbance using Bruker OPUS software. Sodium dodecyl sulphate was used to clean the ATR crystal prior to the first spectral analysis of each sample. The ATR crystal was always completely covered with cells thus simultaneously measuring the maximum number of cells possible. Each spectrum had a background absorption automatically subtracted, was baseline corrected and normalised to amide I absorbance peak (1,650 cm^−1^) using OPUS software. This normalisation function during spectral pre-processing also removed any influence of cell density or absorbance. Average absorbance spectra were then derived using OPUS software, resulting in one absorbance spectrum per sample.

### Statistical analysis

Spectra were processed employing PCA performed using the Pirouette software package (Infometrix Inc., Woodinville, W.A. U.S.A). PCA scores plots were derived and various PCs were examined to see which gave best segregation between cell classes. PCA loadings plots were then derived to determine spectral variance on the selected PCs. PCA was used for preliminary data reduction and the output processed using linear discriminant analysis (LDA) (Fearn, 2002). PCA-LDA scores plots were derived for the biochemical-cell fingerprint region (1,800 cm^−1^ to 900 cm^−1^) and the DNA/RNA region (1,425 cm^−1^ to 900 cm^−1^), with classes assigned as either normal, low-grade or high-grade. PCA-LDA loadings plots for these two spectral regions were also derived (Martin et al. 2007).

## Results

### IR absorbance spectra

**Figure 1:**
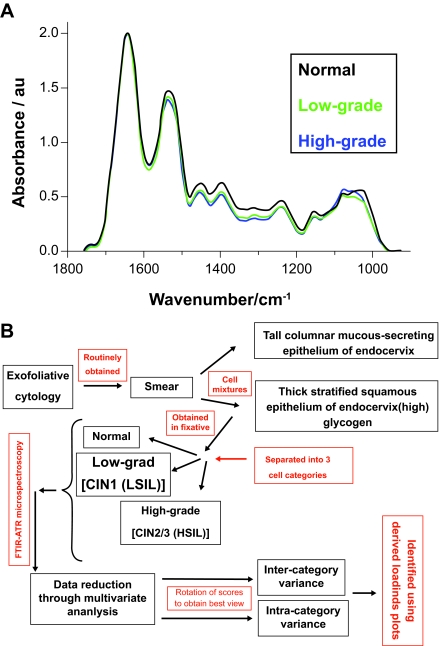
(**A**) Average absorbance spectra of the biochemical-cell fingerprint region (1,800 cm^−1^ to 900 cm^−1^) comparing normal (black), low-grade (green) and high-grade (blue) categories of exfoliative cervical cytology. (**B**) Chronology of the process from the collection of exfoliative cytology smears and identification of histological subtype to the multivariate data analysis.

Figure 1A shows average IR absorbance spectra of the biochemical-cell fingerprint region (1,800 cm^−1^ to 900 cm^−1^) derived from either normal (n = 60), low-grade (n = 60) or high-grade (n = 60) exfoliative cervical cytology. A spectral difference associated with increasing cytology grade was a decrease in intensity of amide II band (1,540 cm^−1^).Associated with increasing severity of atypia, decreases in absorbance intensity are observable throughout the 1,500 cm^−1^ to 1,100 cm^−1^ spectral region; this includes proteins (1,460 cm^−1^), glycoproteins (1,380 cm^−1^), amide III (1,260 cm^−1^), ν_as_PO_2_^−^ (1,225 cm^−1^) and carbohydrates (1,155 cm^−1^). ν_s_PO_2_^−^ (1,080 cm^−1^) appears to have an elevated intensity in high-grade cytology. The glycogen band is prominent in normal smears, but is reduced in the pre-malignant stages (Fig. 1A).

Exfoliative cervical cytology might be categorised into normal, low-grade, high-grade or invasive carcinoma (Fig. 1B). In this study, features responsible for segregation were identified by deriving PCA loadings plots i.e. pseudo-spectra that identify the particular wavenumbers responsible for variance along a particular PC. PCs highlighting inter-category segregation pass through the different categories whereas those which pass through only one category highlight intra-category variance. The data derived from PCA may be further processed using LDA. Here, classes are assigned to the different groupings (e.g. categories of exfoliative cervical cytology). LDA then maximises inter-category variance whilst reducing the influence of intra-class variation (Walsh et al. 2007b).

### PCA of normal *versus* low-grade exfoliative cervical cytology

**Figure 2:**
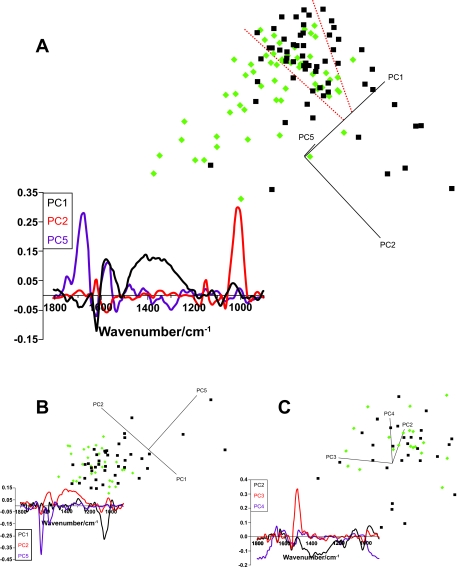
(**A**) PCA scores plot comparing normal (n = 60, black squares) and low-grade (n = 60, green diamonds) categories of exfoliative cervical cytology derived using the biochemical-cell fingerprint region (1,800 cm^−1^ to 900 cm^−1^). The region of overlap between scores for the two categories of exfoliative cervical cytology is identified between the dotted red lines. The corresponding PCA loadings plot which indicates the spectral regions responsible for segregation were derived for PC1 (black), PC2 (red) and PC5 (purple). (**B**) A second PCA scores plot derived from normal (black squares) and low-grade (green diamonds) categories of exfoliative cervical cytology, which were located in the region of overlap or were outliers from the initial PCA run. Loadings plot of the second PCA for PC1 (black), PC2 (red) and PC5 (purple). (**C**) A final scores plot of normal (black squares) and low-grade (green diamonds) categories of exfoliative cervical cytology located in the overlap region or were outliers from the second PCA run. Loadings plot of the final PCA were obtained for PC2 (black), PC3 (red) and PC4 (purple).

Preliminary PCA shows some segregation between normal (n = 60) and low-grade (n = 60) cytology, but a clear region of overlap exists (between dotted red lines) and a number of outlier scores cluster in the “wrong” category (Fig. 2A). Cytology specimens clearly segregated by the derived PCs will account for much of the spectral variance. However, any variance between cytology specimens in the overlap region may be masked. Therefore scores for cytology specimens which clearly segregated were excluded from a further PCA run, with those situated in the overlap region along with outliers being reanalysed to focus in on any variance within such “borderline” specimens (Fig. 2B). This PCA run resulted in an improved discrimination between cell types, with a number of normal scores clearly segregating. Again, absolute segregation of categories was not achieved and a third PCA run was conducted on scores in this overlap region with outliers; this again gave highlighted segregation (Fig. 2C).

Derivation of PCA loadings plots allows the spectral sources of inter- and/or intra-category variance to be identified. This approach of a successive PCA computational model would be expected to allow for the identification of loadings associated with subtle alterations towards discrimination of categories of cytology in overlap regions. In the initial PCA run of normal *versus* low-grade cytology specimens, PC1 is the most important PC for inter-category segregation as it passes through the centre of both normal and low-grade clusters; therefore the variance along this PC is responsible for inter-category segregation (Fig. 2A). The loadings plot highlights the amide I absorbance band (1,650 cm^−1^) and the region 1,500 cm^−1^ to 1,200 cm^−1^. Successive PCA of normal *versus* low-grade cytology specimens designates PC5 as important; this again points to the importance of the amide I absorbance band (Fig. 2B). Following a third PCA run, PC2 is shown to give rise to segregation and the derived loadings plot again highlights the region 1,500 cm^−1^ to 1,200 cm^−1^ as responsible (Fig. 2C). Therefore, spectral bands associated with amide I (1,650 cm^−1^) and 1,500 cm^−1^ to 1,200 cm^−1^ are consistently important for inter-category segregation of normal and low-grade exfoliative cervical cytology.

Sources of intra-category variance are demonstrated by PC2 in the initial PCA run (Fig. 2A), as it is responsible for “spreading” both normal and high-grade scores; PC2 is strongly associated with the glycogen absorbance band (1,030 cm^−1^). This is re-enforced by PC1 in the second PCA run. In the final PCA run, the derived loadings plot for PC3 suggests the amide II shoulder (1,500 cm^−1^) as a source of intra-category variance.

### PCA of normal *versus* high-grade exfoliative cervical cytology

**Figure 3:**
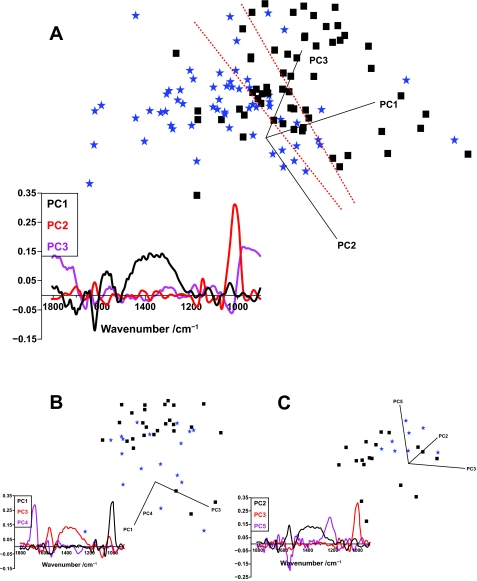
(**A**) PCA scores plot comparing normal (n = 60, black squares) and high-grade (n = 60, blue stars) categories of exfoliative cervical cytology derived using the biochemical-cell fingerprint region (1,800 cm^−1^ to 900 cm^−1^). The region of overlap between the scores for the two cell categories is identified between the dotted red lines. Corresponding PCA loadings plot which indicates the spectral regions responsible for segregation were derived for PC1 (black), PC2 (red) and PC3 (purple). (**B**) A second PCA scores plot derived from specimens that were located in the region of overlap or were outliers from the initial PCA run. Loadings plot of the second PCA for PC1 (black), PC3 (red) and PC4 (purple). (**C**) A final scores plot of specimens located in the overlap region or were outliers from the second PCA. Loadings plots of the final PCA were obtained for PC2 (black), PC3 (red) and PC5 (purple).

Initial PCA of normal *versus* high-grade exfoliative cervical cytology showed discrimination between the two categories (Fig. 3A). Again, a successive PCA was performed on the region of overlap identified between the dotted red lines. The second PCA run gave rise to good segregation of scores derived from both categories of cervical cytology; however, three apparently normal scores fell within the high-grade cluster (Fig. 3B). A third PCA run on the region of overlap with outliers in the second PCA demonstrated clear discrimination with clustering of scores derived from high-grade specimens (Fig. 3C).

Loadings plots of the three successive PCA run were derived to identify the spectral bands responsible for inter-category variance between normal and high-grade. PC1 and PC3 highlighted the contributions of 1,800 cm^−1^, 1,610 cm^−1^, 1,500 to 1,200 cm^−1^ and 970 cm^−1^ to inter-category discrimination (Fig. 3A). The second PCA run did not suggest a particular PC as contributing to either inter- or intra-category discrimination (Fig. 3B). In the final PCA run, PC2 was responsible for inter-category segregation with amide II (1,550 cm^−1^) and the 1,500 cm^−1^ to 1,200 cm^−1^ region being responsible (Fig. 3C). Intra-category variance associated with glycogen (1,030 cm^−1^) was highlighted by PC2 in the initial analysis (Fig. 3A) and PC3 in the final comparison (Fig. 3C).

### PCA of low-grade *versus* high-grade exfoliative cervical cytology

**Figure 4:**
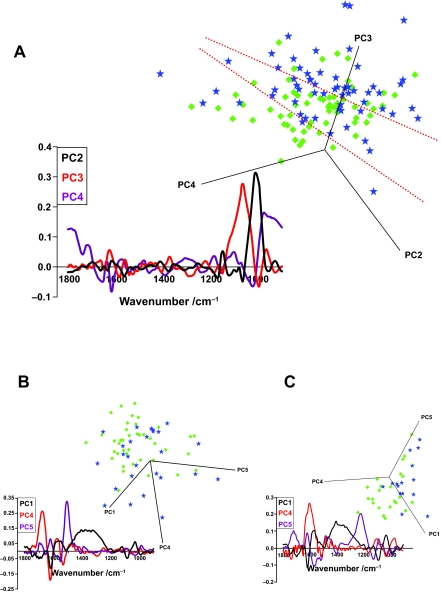
(**A**) PCA scores plot comparing low-grade (n = 60, green diamonds) and high-grade (n = 60, blue stars) categories of exfoliative cervical cytology derived using the biochemical-cell fingerprint region (1,800 cm^−1^ to 900 cm^−1^). The region of overlap between the two cell categories is identified between the dotted red lines. Corresponding PCA loadings plot which indicates the spectral regions responsible for segregation were derived for PC2 (black), PC3 (red) and PC4 (purple). (**B**) A second PCA scores plot derived from specimens that were located in the region of overlap or were outliers from the initial PCA. Loadings plot of the second PCA for PC1 (black), PC4 (red) and PC5 (purple). (**C**) A final scores plot of specimens located in the overlap region or were outliers from the second PCA. Loadings plots of the final PCA were obtained for PC1 (black), PC4 (red) and PC5 (purple).

Initial PCA comparison of low-grade and high-grade showed some segregation between these two categories of cervical cytology (Fig. 4A). Again, in the scores plot a region of overlap with a number of outliers is observed; these scores were subjected to further PCA, giving rise to improved segregation (Fig. 4B). A third PCA run between these low-grade and high-grade categories of cervical cytology resulted in good separation (Fig. 4C). In the initial PCA, PC3 was most responsible for segregation of the two categories of cytology, with ν_s_PO_2_^−^ (1,080 cm^−1^) being most prominent (Fig. 4A). Intra-category variance was highlighted by PC2 and pointed to glycogen (1,030 cm^−1^). In the second PCA run, PC4 highlighted 1,670 cm^−1^ and 1,630 cm^−1^ associated with the amide I peak (as did the final PCA run) and 1,540 cm^−1^ associated with the amide II peak (Fig. 4B and 4C). Towards intra-category variance, PC5 in the second PCA run pointed to the amide II shoulder (1,500 cm^−1^). In the final PCA, PC1 and PC5 highlighted the influence of 1,610 cm^−1^, the 1,500 cm^−1^ to 1,200 cm^−1^ region and ν_as_PO_2_^−^ (1,225 cm^−1^) (Fig. 4C).

### PCA-LDA of cervical cytology categories

**Figure 5:**
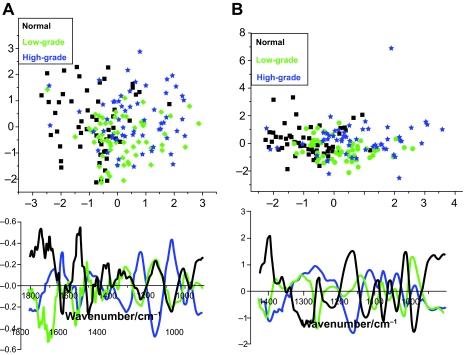
PCA-LDA scores plot using the (**A**) biochemical-cell fingerprint region (1,800 cm^−1^ to 900 cm^−1^) or (**B**) the DNA/RNA region (1,425 cm^−1^ to 900 cm^−1^) with classes assigned as normal (n = 60, black squares), low-grade (n = 60, green diamonds) or high-grade (n = 60, blue stars) categories of exfoliative cervical cytology. PCA-LDA loadings plots were derived for normal (black), low-grade (green) and high-grade (blue), to demonstrate the spectral differences between the average spectrum of each category compared to the average spectrum of all categories.

All the specimens of exfoliative cervical cytology (n = 180) were assigned classes according to histological grading [i.e. normal, low-grade or high-grade (n = 3)] and PCA-LDA was carried out. PCA-LDA scores plots were derived using either the entire biochemical-cell region (1,800 cm^−1^ to 900 cm^−1^) or the DNA/RNA region (1,425 cm^−1^ to 900 cm^−1^). The scores plots of the biochemical-cell region showed some degree of segregation between normal (n = 60), low-grade (n = 60) and high-grade (n = 60) categories (Fig. 5A). Normal and high-grade cytology categories segregated, with low-grade scores seemingly positioned in the middle. The PCA-LDA scores plot of the DNA/RNA spectral region appears to give improved segregation between cell types, with a seemingly clear progression from normal through to low-grade to high-grade being demonstrated (Fig. 5B).

The aim of employing PCA-LDA is to reduce the influence of intra-category variation and maximise inter-category variance as these loadings plots are derived for each category. The loadings are the spectral changes discriminating the average spectrum of one category from the average of spectra from all three categories. Resultant loadings of the biochemical-cell region show that the spectral bands that distinguish normal and low-grade are similar, with contributions from the 1,800 cm^−1^ to 1,650 cm^−1^ region, 1,550 cm^−1^, 1,500 cm^−1^, 1,425 cm^−1^, 1,155 cm^−1^ and 1,080 cm^−1^ (Fig. 5A). Loadings for high-grade point to 1,610 cm^−1^, 1,300 cm^−1^, 1,155 cm^−1^ and 1,080 cm^−1^. The loadings plots derived from the PCA-LDA analysis using the DNA/RNA region show that spectral contributions for normal are 1,400 cm^−1^, 1,300 cm^−1^ and 1,155 cm^−1^, for low-grade 1,250 cm^−1^, and for high-grade 1,250 cm^−1^ and 1,155 cm^−1^. The 1,150 cm^−1^ to 900 cm^−1^ region is found to be an important contributor for segregation of all cell types.

### Intra-category variance in low-grade cervical cytology

**Figure 6:**
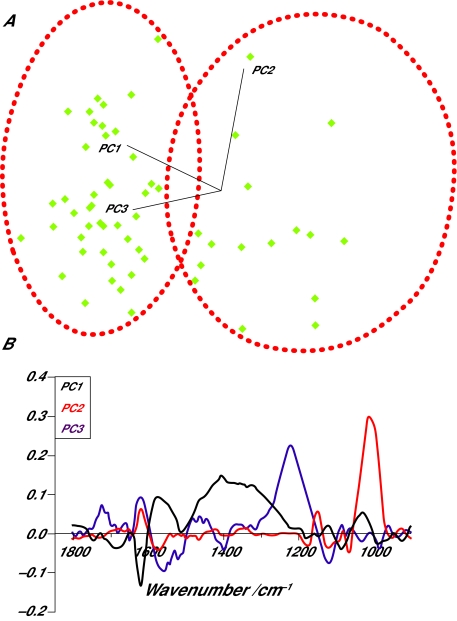
(**A**) PCA scores plot using the biochemical-cell fingerprint region (1,800 cm^−1^ to 900 cm^−1^) of low-grade specimens (n = 60, green diamonds) to identify apparent CIN1 subgroups. (**B**) A corresponding PCA loadings plot derived for PCs 1 (black), 2 (red) and 3 (purple).

PCA of low-grade specimens (n = 60) appears to show two distinct clusters. The majority of the low-grade specimens (n = 43) clustered together whereas a small subset of low-grade cytology specimens (n = 17) clustered separately (Fig. 6A). Derived loadings demonstrated that PC1 and PC3 were responsible for segregation of these putative clusters, and pointed to spectral variance at wave-numbers 1,610 cm^−1^, 1,550 cm^−1^, 1,500 cm^−1^ to 1,200 cm^−1^ region and ν_as_PO_2_^−^ (1,225 cm^−1^). Intra-category variation, determined most by PC2, was associated with glycogen (1,030 cm^−1^) (Fig. 6B).

## Discussion

Different categories of exfoliative cervical cytology that had previously been categorised based on visual smear cytology were obtained. Following interrogation using ATR microspectroscopy, derived IR spectra were subjected to PCA. Two categories at a time were coupled with successive PCA of overlapping regions with outliers to identify the potential variance between these cell classes i.e. normal *versus* low-grade or high-grade and low-grade *versus* high-grade.

These analyses resulted in discrimination between different cell classes. Clusters for normal *versus* low-grade showed the least clear segregation; whether this is to be expected as some low-grade cervical lesions often regress to normal remains to be ascertained. However, this suggested that the biochemical progression from normal to low-grade is less definitive than from low-grade to high-grade. Exfoliative cervical cytology cate-gorised as low-grade by conventional visual screening is often left untreated, with the proviso that follow-up smears are required. The likelihood of CIN1 regression to normal is ≈60%, with 30% persisting as CIN1, 10% progressing to CIN2/3 and 1% progressing to invasive carcinoma (Ostor, 1993). Visual examination of the morphological characteristics of CIN1 cervical cytology specimens remains limited with regards to predicting outcome (Melnikow et al. 1998).

IR spectra derived from CIN1 specimens were subjected to PCA; examination of the scores plot appears to suggest clustering into two distinct groups (Fig. 6A). The scores clustered on the right are the same ones that segregate furthest from the scores for normal cytology in the normal *versus* low-grade comparison (Fig. 2A). These are also the same scores that cluster closest to the scores for high-grade cytology in the low-grade *versus* high-grade comparison (Fig. 3A). These observations may point to a novel approach to segregating CIN1 exfoliative cervical cytology on the basis of whether it may progress or whether it may regress without the necessity for intervention.

Examination of vibrational spectra following IR microspectroscopy may also facilitate the identification of molecular or chemical alterations responsible for either inter- or intra-category variance. Such chemical groupings that appear important for segregation of cytology categories, consistently indicated in loadings plots following PCA or PCA-LDA, are the amide I absorbance peak (1,670 cm^−1^ to 1,630 cm^− 1^) and the 1,500 cm^−1^ to 1,200 cm^−1^ region. The identification of amide I as an important biomolecule for segregation of categories of cervical cytology demonstrates the computational power of PCA; the average IR spectra show apparently little change in amide I peak between different classes (Fig. 1A).

The greatest source of intra-category variance is consistently associated with glycogen levels (1,030 cm^−1^) and to some extent, the amide II shoulder (≈1,500 cm^−1^). PCA-LDA reduces the sources of intra-category variance; when PCA-LDA loadings plots were derived, there was an absence of a glycogen contribution to variance (Fig. 5A). Glycogen levels in exfoliative cervical cytology may be an important contributor to intra-category variation as a consequence of differences in various sampling techniques. The smear-taking process involves sampling cells in the transformation zone, which is located between the endocervical canal and ectocervical zone (Fig. 1B). As squamous endocervical cells mature through intermediate and superficial layers their levels of intra-cellular glycogen increase, whereas columnar endocervical cells do not store glycogen (Chiriboga et al. 1998; Romeo et al. 2002). Intra-category variance may be a consequence of variation in the proportions of these two cell types. Cyclical changes associated with the menstrual cycle may also alter the IR absorbance spectra of ectocervical cells (Romeo et al. 2002).

IR microspectroscopy with subsequent multivariate analysis of cytological specimens could allow for an objective classification approach, with cell classes designated by a panel of chemical groupings which would allow for more accurate categorisation, in particular for borderline cases. However, long-term follow-up of low-grade exfoliative cervical cytology may be required to determine the usefulness of this approach.
